# ARM in ARM? Investigating the co-occurrence of anorectal malformations and labioscrotal anomalies

**DOI:** 10.1007/s00383-026-06475-7

**Published:** 2026-07-17

**Authors:** Patrick G. G. Sharman, Maria Randazzo, Ian Jones, Ingo Jester

**Affiliations:** 1 Medical School, College of Medicine and Health, University of Birmingham, Edgbaston, B15 2TT UK; 2 Università degli Studi di Milano-Bicocca, Piazza dell’Ateneo Nuovo 1, 20126 Milan, Italy; 3 Birmingham Women and Children’s NHS Foundation Trust, Steelhouse Lane, Birmingham, B4 6NH UK

**Keywords:** Anorectal malformation, Labioscrotal anomaly, Multiple congenital malformations, Embryology

## Abstract

Anorectal malformations (ARMs) are known to be associated with other defects, however there is no evidence addressing the co-occurrence of ARMs with anomalies of structures derived from the labioscrotal folds. We performed a systematic review (Prospero: CRD420251013945) aiming at investigating correlations and patterns between ARMs and labioscrotal anomalies (LSAs), when presenting simultaneously. Our search identified 1642 articles. After abstract screening, 344 full texts were assessed and 144 met the inclusion criteria. In total, these reported on 319 individual cases with both ARM and LSA. Individuals with complex ARMs had proportionally the most LSAs associated with their ARM − 58.4% of these were associated with only a single LSA. For ‘high’ and ‘low’ ARMs, 66.0% and 77.0% of cases were associated with a single LSA respectively. As data reporting prevalence of each type of ARM lacks information about LSAs, the potential to predict the presence of LSAs according to type of ARM is limited. Nevertheless, this review demonstrates a frequent association of ARMs with LSAs, that has not been previously described. This evidence highlights the importance of early screening and timely management of LSAs in patients with any type of ARM, with close collaboration between paediatric surgeons, urologists and other clinicians.

**Supplementary Information:**

The online version contains supplementary material available at 10.1007/s00383-026-06475-7.

## Introduction

Anorectal malformations (ARMs) represent a spectrum of congenital defects of the anus and rectum, ranging from anal stenosis to severe anatomical abnormalities such as a persistent cloaca. Often, they can be associated with the presence of additional abnormalities, including labioscrotal anomalies (LSAs). Their association with certain other distinct anatomical defects is well established [1]. Early studies reported that between 49.4% and 71.0% of infants had other anomalies associated with their ARM [1–3]. More recent cohort studies, using the updated Krickenbeck criteria [4], suggest the rate is much higher; Oh et al. found 65.0% of 460 patients (*n* = 299) with an ARM had at least one associated defect, and major anomalies were found in 59.6% (*n* = 274) [5]. Much of this research has considered the association of urologic and reproductive abnormalities with ARMs [6, 7], consequently the management of infants with such a presentation is well-established [8]. However, established approaches to the management of infants with genitourinary anomalies associated with their ARM has not reconciled the different embryological origin of the labia majora and scrotum to the rest of the urogenital system as a potential source of a need for different approaches to management.

‘Labioscrotal’ refers to a bipotential embryonic structure which develops into the scrotum in genetic males, and the labia majora in genetic females [9]. It is of distinct embryological origin to the rest of the urogenital system [9–12]; aberrations of the developmental process give rise to anatomical anomalies specific to the scrotal and labial structures. Whilst there is currently no classification or systematic nomenclature to describe these LSAs, a variety of anatomical malformations affecting the structures derived from the labioscrotal folds have been reported [13–29]. These include bifid scrotum, ectopic scrotum, accessory scrotum, hemiscrotum, penoscrotal fusion, penoscrotal transposition, scrotal raphe anomaly, scrotoschisis, (hemi)scrotal agenesis/hypoplasia, proximal hypospadias, labial hypoplasia, labial hypertrophy, labial fusion and accessory labial fold [29, 30]; a brief summary of the anatomy and theorised embryological basis for these LSAs is provided in Supplementary Table 1. Because of their different embryological origin to the rest of the urogenital system, it is possible that LSAs are caused by aberrations with a distinct pathogenesis to those of the rest of the urogenital system.

A recent multi-country study found the pooled prevalence of ARMs to be 3.26 per 10,000 births [30]. Concerning the incidence of LSAs, there is a lack of consensus about how they should be described and diagnosed, meaning that there is very little data on their incidence as isolated defects. Therefore, it is not surprising that the co-occurrence of ARM and LSA appears to be absent from existing literature entirely. As more severe ARMs have poorer outcomes [31–33], and are more commonly associated with other congenital defects [1, 5, 33], the logical conclusion would be to assume that the same is true of any relationship which may exist between ARMs and LSAs. However, the paucity of data means that this has by no means been established.

This review aims to verify the existence of such a relationship between ARMs and LSAs as a distinct group of defects, in terms of possible embryological correlations and patterns of presentation. The clinical implications of various ARM-LSA associations are considered as well, in particular the importance of early screening for LSAs in neonates presenting with ARMs, the short- and long-term consequences and management of these patients, and the need for close collaboration between different medical disciplines.

## Materials and methods

A search strategy was developed (Prospero: CRD420251013945) to interrogate the various databases, that took account of the fact that there are no current MeSH terms for LSAs (search terms used by authors for ARMs and LSAs in Supplementary Table 2). MEDLINE, Embase, Cochrane, CINAHL Plus EBSCO and ProQuest were all searched, with no restrictions on publication date. Searches were performed from 15/04/2024–16/04/24. Forwards and backwards reference checking of included studies was continued up until the data synthesis stage (17/07/25). All authors of cohort studies which did not give individual-specific data were contacted for further information about each patient’s defects. Journals containing studies that were identified electronically during scoping searches but that were only accessible as physical copies were also hand-searched.

Clarivate EndNote Online was used to combine and store the results of the literature searches [34]. Papers were screened by two independent assessors (PS and MR), with any conflicts resolved by discussion with IJ1 and IJ2. Studies were included if they provided data of examples of co-occurrence of a specific ARM and a specific LSA, for example anal stenosis alongside bifid scrotum. Foreign language articles were included; translators and translation software were used when necessary. The lack of restriction on publication date allowed for reports using outdated terminology to be included, provided that sufficient description of the anatomy was given to enable a specific ARM and specific LSA to be identified. Conference abstracts or poster presentations were included as full text articles if relevant information was included within the text available; efforts were made to confirm that data obtained from these had not been duplicated in full text reports, for example checking that multiple records with the same author were indeed reporting independent cases.

Papers were excluded if they: reported an ‘anorectal malformation’ with no further detail of the type of malformation; reported co-occurrence of hypospadias with ARM without specifying the type of hypospadias; reported anteriorly placed anus as an ARM (with no other defect); reported acquired ARM-LSA; or reported defects of the external genitalia with no further detail.

Data extracted from eligible papers included genetic sex of patient (phenotypic sex was not always clear in cases of ambiguous external genitalia), type of ARM, and type of LSA diagnosed. Data was tabulated using Microsoft Excel [35] (Tables 2, 3 and 4).

The type of ARM provided headings for the columns (using Krickenbeck nomenclature [4]); additional ARM headings were included for ‘rectobladder neck fistula’, ‘unspecified rectourethral fistula’ and ‘unspecified ARM’ (the latter referring to cases where enough detail was provided that the defect could be confirmed to be an ARM, but not enough detail that a specific label could be assigned). Type of LSA provided the headings for the rows; these were based on the terms used for the literature search (Supplementary Table 2). The total number of cases of each type of ARM was recorded, as well as the total number of LSAs identified per type of ARM (more than one LSA could be associated with the same patient’s ARM).

The wide range of categories in the Krickenbeck classification and the rarity of ARM-LSA means that there were too few cases of ARM-LSA co-occurrence for patterns to be suggested by each type of ARM on its own. Therefore, the ARMs were grouped into ‘low’, ‘high’ and ‘complex’, to produce larger subgroups of individuals whose data can produce analyses of greater power. Whilst it is not ideal to combine classification systems in this way, the authors argue that the benefit of a more robust statistical analysis outweighs such disadvantages.

This necessary grouping is based on historic classifications of ARMs [36–38]; ‘low’ and ‘high’ refer to the level of the fistula/defect in relation to the levator ani muscle, with the latter being recognised as more severe. ‘Complex’ is used to distinguish defects such as cloaca, pouch colon, H-type fistula and ‘no fistula’, which are yet more severe and require greater intervention. The ARMs assigned to each group are shown in Table 1. Cases describing an LSA that were assigned to ‘unspecified ARM’ or ‘other’ were recorded for the sake of completeness, but were not included in the ‘low’/‘high’/complex grouping, nor were they included in subsequent data analysis.

**Table 1:** Grouping of ARMs based on severity

‘Low’ ARMs	‘High’ ARMs	Complex ARMs
Perineal (cutaneous) fistula Vestibular fistula Rectovaginal fistula Anal stenosis	Rectobladder neck fistula Rectourethral bulbar fistula Rectourethral prostatic fistula Unspecified rectourethral fistula Rectovesical fistula Rectal atresia	Cloaca Pouch colon H-type fistula No fistula

## Results

### Literature search

**Fig. 1:**
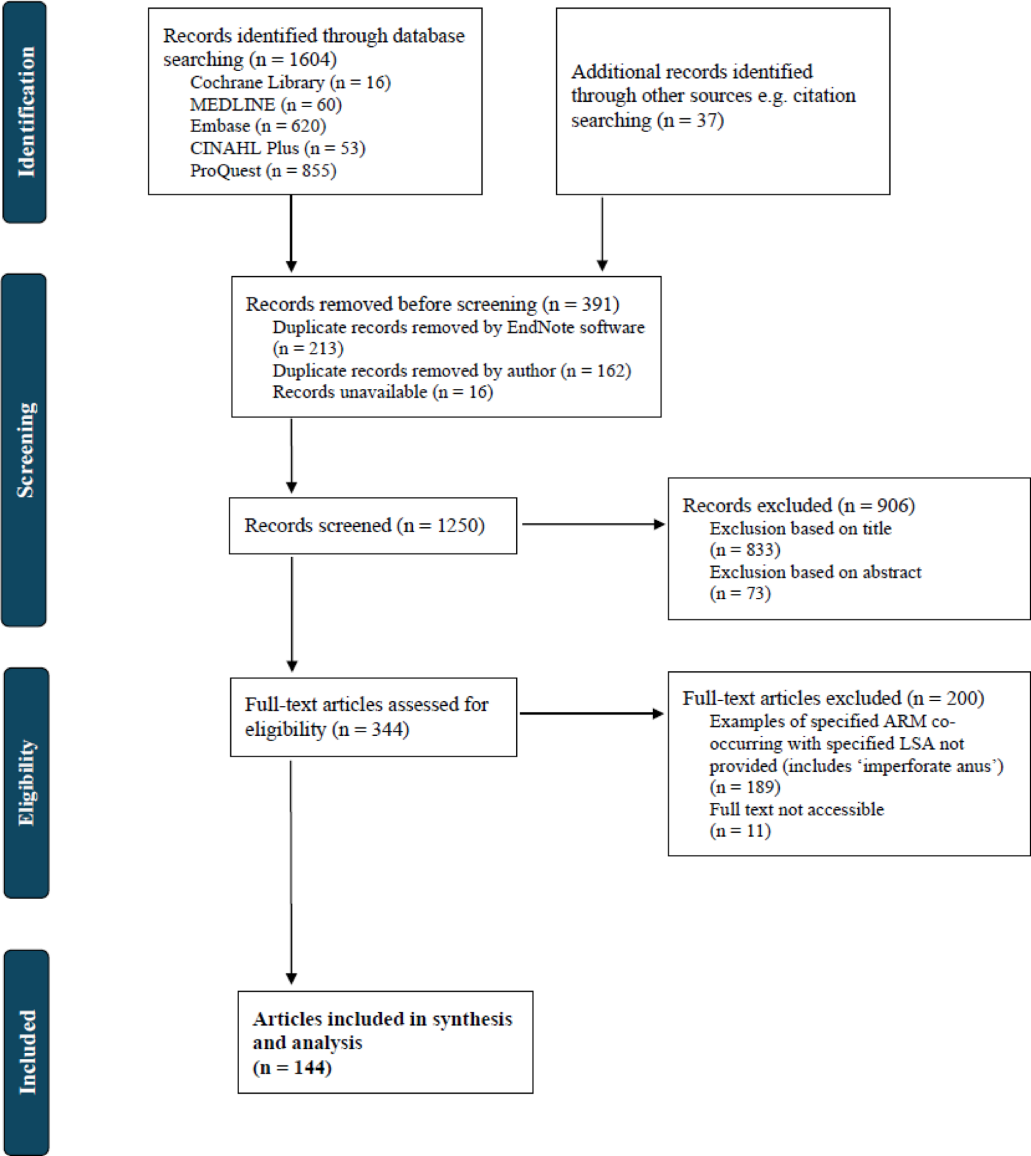
PRISMA-style flowchart of literature searches and screening process [39]

The results of the database searches and subsequent screening are displayed in a PRISMA-style flowchart (Fig. 1) [39]. The searches from the five databases that were used produced 1604 articles. 375 duplicates were identified and removed; 16 articles were excluded because their full texts were not accessible and sufficient information was not available from their abstracts for analysis. A further 37 records were identified after the literature search. In total, 1250 abstracts were screened, of which 144 met the inclusion criteria.

### Data extraction

From the 144 full-text records accepted, 319 individuals were identified with an ARM co-occurring with one or more LSA (Table 2); 261 of these were genetic males and 58 were genetic females. In males, the highest incidence of individual cases of ARM associated with one or more LSA was with perineal (cutaneous) fistula (*n* = 40). The highest incidence of individual cases of ARM co-occurring with one or more LSA in females was cloaca, found in 26 patients.

**Table 2:**
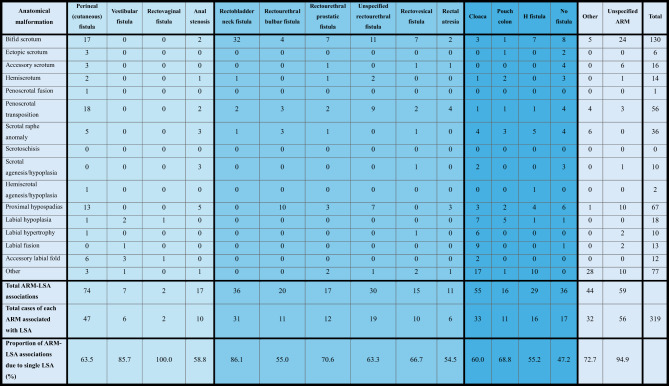
All cases of ARM-LSA co-occurrence

### Males

In genetic males (Table 3), there were no cases of rectovaginal fistula co-occurring with LSA, nor were there any cases of scrotoschisis, labial hypoplasia or labial fusion associated with an ARM. Of note was the 31 separate cases of ‘Other’ ARMs associated with an LSA in males, of which the majority were recto- or anoscrotal fistulae; 9 cases of recto- or anoscrotal fistulae came from a single case series [40].

**Table 3:**
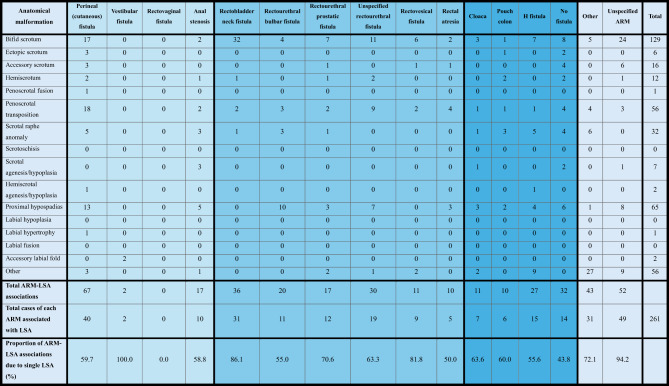
Cases of ARM-LSA co-occurrence in males

### Females

There were no cases of rectourethral fistula (of any type) or rectobladder neck fistula associated with an LSA in genetic females (Table 4). Scrotal anomalies were very rare in genetic females. There was at least one instance of each type of labial anomaly (labial hypoplasia, labial hypertrophy, labial fusion, accessory labial fold) co-occurring with an ARM.

**Table 4:**
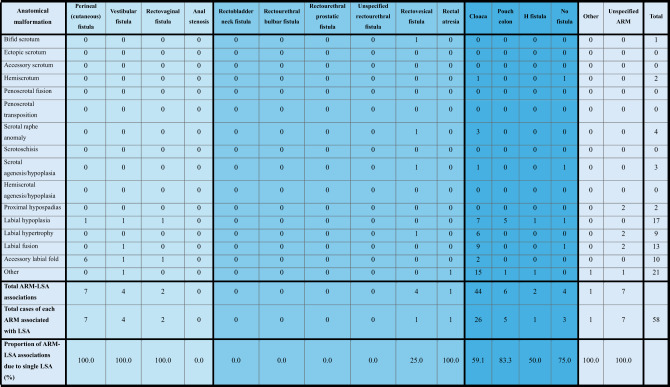
Cases of ARM-LSA co-occurrence in females

### Further analysis

Data from males and females were combined, and the total numbers of patients and associated LSAs found for each type of ARM was calculated. To identify and compare which types of ARM tended to have multiple LSAs co-occurring in a single patient, the number of patients with a given type of ARM (all of whom had at least a single LSA) was calculated as a proportion of the total number of ARM-LSA associations found for the same type of ARM (Table 2). This showed that ‘no fistula’ had the greatest number of LSA associations relative to the number of patients with these ARMs: 47.2% of individuals with ‘no fistula’ had only a single LSA associated with their ARM. The ARM with the smallest difference between number of patients and number of associations was rectovaginal fistula, where every patient had only a single LSA associated with their ARM (= 100.0%).

**Fig. 2:**
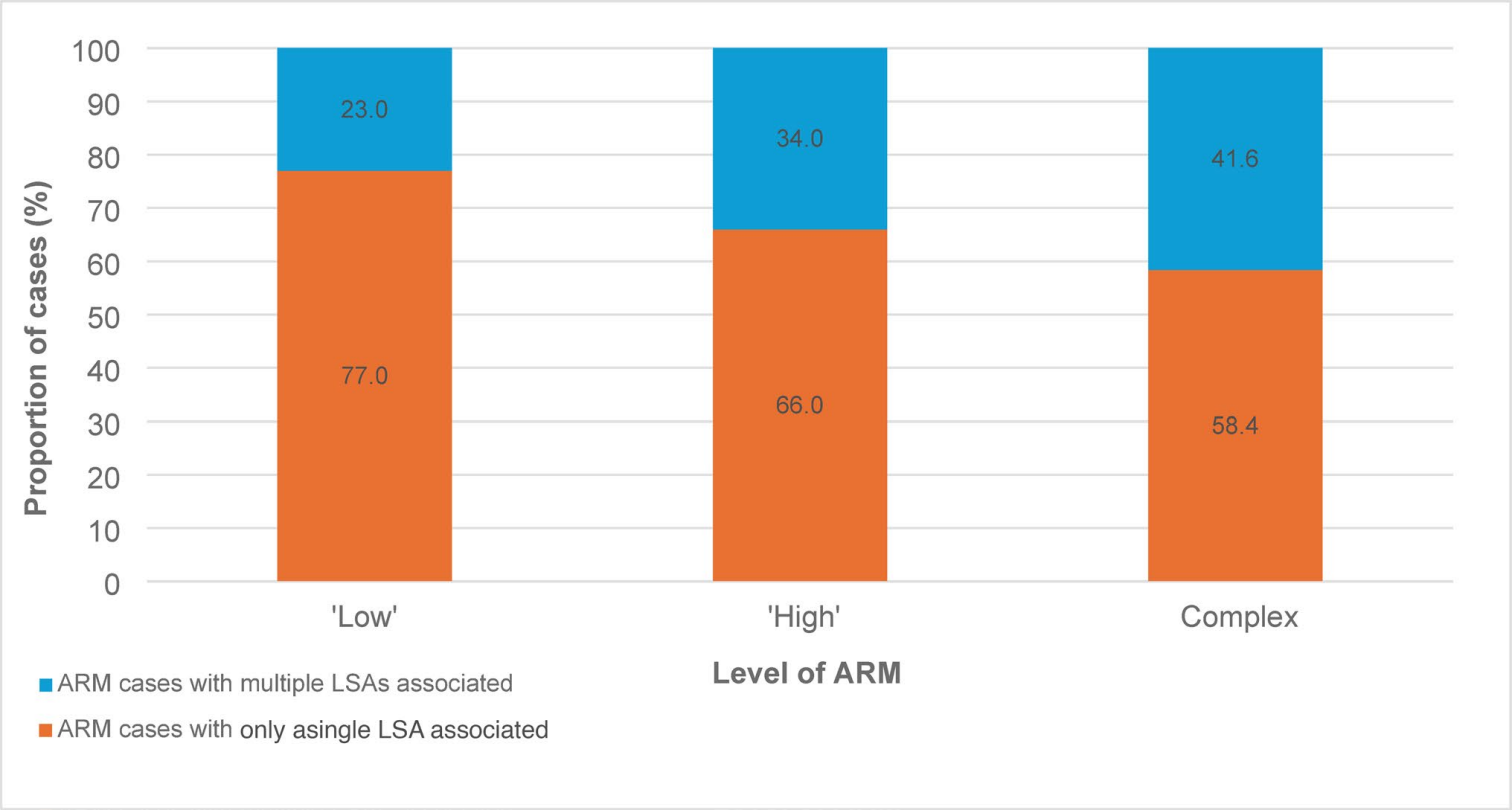
Proportion of ARM cases associated with single vs. multiple LSAs (‘low’, ‘high’, complex ARMs)

This approach was taken for all types of ARM, then averaged into groups based on severity of ARM: ‘low’/‘high’/complex (Table 1). These calculations excluded the datasets for ‘Other’ and ‘Unspecified ARM’, as the level of ARM could not be verified for these patients. Figure 2 demonstrates that the proportion of complex ARM cases associated with multiple LSAs (= 41.6%) was higher than that for ‘high’ ARM cases (= 34.0%), which itself was higher than the proportion of ‘low’ ARM cases associated with multiple ARMs (= 23.0%). Viewed in terms of associations with a single ARM, Fig. 2 shows that the proportion of ARMs associated with only a single LSA decreased as severity of ARM increased. For further illustration of this trend, see Supplementary Fig. 1.

## Discussion

No previous study in the literature has looked systematically at the potential association of ARMs with LSAs without grouping LSAs under the generic label of ‘genitourinary anomalies’ (with the exception of Wang et al.’s case series investigating the triple co-occurrence of ARM, LSA and perineal lipoma [29]). This review has demonstrated that, per patient, those with more severe ARMs are more likely to have a greater number of LSAs simultaneously present. This finding therefore suggests that there is likely to be a link between ARMs and LSAs that needs further study: 319 instances of ARM-LSA association were identified, across the spectrums of both ARMs and LSAs. In this way, our findings also demonstrate that ARMs, regardless of severity, may co-occur with at least one LSA with a frequency that warrants further investigation. Given that the clinical burden of congenital malformations increases with the number of anomalies [41, 42], a comparison between the number of individuals documented and the total number of LSAs recorded across these individuals was considered a suitable method to determine whether some ARMs were proportionally associated with more LSAs than others. As shown by Fig. 2, as the severity of ARMs increases, the difference between the number of individuals recorded and the total number of LSAs recorded also increases; i.e. a patient with a complex ARM is likely to have more LSAs associated with their ARM than a patient with a ‘high’ ARM, who themselves is likely to have more LSAs than a patient with a ‘low’ ARM.

Ideally, a gradient of ‘LSA association’ for the whole spectrum of ARMs would have been produced, which could demonstrate whether one specific type of ARM is more likely to have more LSAs than another. However, there were very few cases of ARM-LSA association for some malformations, for example only two cases of rectovaginal fistula and only six of vestibular fistula co-occurring with LSAs. Therefore, due to the paucity of data at this point, it was not possible to produce meaningful analyses of individual-ARM-level and LSA association. The apparent absence of similar studies that may have produced results with which ours can be compared presents a challenge in verifying the outcomes discussed above. Indeed, without similar studies or further epidemiological data on ARMs and LSAs, it cannot be said with certainty whether the observed patterns of certain LSAs co-occurring with certain ARMs are due to chance, or the result of a genuine link between the two groups of malformations, be it of embryological, genetic or physiological origin. For example, based on the data in Table 2, one might predict rectobladder neck fistulae to co-occur most commonly with bifid scrotum, or that bifid scrotum is the LSA that is most likely to be associated with rectobladder neck fistulae (*n* = 32). However, bifid scrotum may be a particularly common defect, independent of the presence of an ARM, when compared to other LSAs - this would seem to be the case based on its wider reporting in existing literature [13, 43, 44]. Alternatively, this review may have missed substantial datasets which report the co-occurrence of bifid scrotum with another ARM. For example, the majority (*n* = 18) of the rectobladder neck fistula/bifid scrotum associations came from a single cohort collected by Peña and Bischoff, which may have chosen to specifically report this association [45]. The most useful information for the present study would be statistics which report the prevalence of LSAs occurring without ARMs, to identify whether bifid scrotum has been reported frequently because it is a relatively frequent labioscrotal defect, or because it is a defect which is particularly likely to arise when there is also an ARM.

Whilst the frequency of LSAs’ co-occurrence with ARMs compared to their incidence in isolation cannot be commented on, the recent development and release of the ARM-Net database [46] may provide a means of analysis from the reverse point of view. ARM-Net is the first large-scale database which reports the prevalence of each individual type of ARM, according to the Krickenbeck classification [4]. By comparing the database’s information on the incidence of each type of ARM in isolation from LSAs with the results of *this* study, initial insights into the frequency with which ARMs co-occur with LSAs, compared with their occurrence without LSAs may be gained. It is important to note that this comparison would require two considerable assumptions to be made: firstly, that enough cases of all combinations of ARM-LSA co-occurrence to be representative of their true prevalence have been documented; secondly, that this systematic review has successfully identified all of these cases. As rigorous as this study has attempted to be in its searches, neither assumption can be reasonably accepted for the purposes of informing epidemiological data.

Even without the necessary data available to analyse patterns of co-occurrence at the LSA-specific or ARM-specific level, this review’s findings of the number of LSAs increasing with increasing severity of ARM suggests that the embryological relationship of the two groups of defects should be examined. The shared cloacal origin of the anorectum and genitourinary tract has been the source of much investigation regarding ARMs and associated urological anomalies [3, 10, 24, 47–57]; molecular studies by Sun et al. have even demonstrated that knockout of the signalling molecule Dickkopf-1 (normally expressed in the dorsal/posterior cloaca, which gives rise to the anorectum) in mice resulted in linked anorectal and urologic anomalies, via the upregulation of the *Wnt/*β-catenin pathway, sonic hedgehog, fibroblast growth factor 8 and bone morphogenetic protein 4 [55]. Earlier work comparing signalling pathways relating to hindgut development in mice and humans [47, 58, 59] has demonstrated that mouse models behave sufficiently similarly to human embryological processes that mechanisms such as those observed in mice by Sun et al. [55] may be used to suggests hypotheses about abnormal development in humans. However, whilst these studies may provide insights into the aetiology of many genitourinary anomalies associated with ARMs, they do not acknowledge how the independent formation of the labioscrotal structures may be disrupted, and how this might be related to the disruption of anorectal structures.

Indeed, it is unclear at this point whether the co-occurrence of ARMs and LSAs is due to an embryological mechanism (like that demonstrated by Sun et al.) which specifically affects both the development of the posterior cloaca and the labioscrotal folds, or due to a more systemic developmental problem. The anorectum and labioscrotal folds do not share an exact histological origin, as the anorectum and other genitourinary structures do: the anorectum, bladder and most of the urethra originate from dense pericloacal mesenchyme and endoderm, whereas the labioscrotal folds are derived from loose mesenchyme and ectoderm [9–12, 60]. Research to distinguish patients with ARM-LSA co-occurrence only, from those with ARM-LSA as well as other associated anomalies, is required to understand this.

Although much of the focus of this systematic review has been to identify an embryological correlation between ARMs and LSAs, our review of the literature has also demonstrated the overall rarity of this presentation. This makes it difficult to provide the clinician with a robust treatment strategy; best timing of surgical management is not clear in the absence of a classification and standardised treatment. The paediatric surgeon’s immediate priority is likely to focus on resolving the problems caused by the ARM, and whilst problems of faecal (in)continence and constipation may be controlled in childhood, Bjoersum-Meyer et al.’s systematic review of ARM patients’ long-term outcomes demonstrates that urinary continence often remains an issue in later life, as well as cosmetic, sexual and gynaecological problems which only arise post-puberty [61]. It is likely that these problems are compounded when an LSA is present alongside the ARM; unfortunately the lack of long-term follow-up research prevents an evidence-based recommendation for management from being made. Nevertheless, it is clear that the timing of the repairs of the ARM and the LSA should not be seen in isolation from each other: early multi-disciplinary collaboration is critical to improve all patient outcomes. Considering the short- and long-term challenges that patients with ARMs and LSAs may face (including urinary incontinence, erectile dysfunction, ejaculatory dysfunction, infertility, psychosocial and behavioural development [62–66]), a holistic approach to management from the time of diagnosis until discharge from paediatric care is advised to achieve the best outcomes for continence, fertility, sexual function, cosmesis and psychological health.

### Limitations

Performing a systematic review to investigate anatomical abnormalities was a complex challenge, as the nature of the data sought made the process different to a standard systematic review. This placed limitations on the methodology which may have reduced the reliability and completeness of this review. Furthermore, due to increasing pressure on publications to gain citations, and restrictions on acceptance from journals, it is becoming increasingly difficult to publish case reports [67]. As a result, it is highly likely that these rare cases of ARM-LSA association are being operated on without being reported – therefore the correlation of these anomalies may be even stronger than that demonstrated by this review. This emphasises the need for an international database for the reporting of these defects, which could form the foundation of specific clinical guidance on the management of ARMs associated with LSAs.

Use of vague or historic nomenclature [36–38] excluded a large number of records, for example a significant proportion only described the ARM as ‘imperforate anus’. This and other misused terminology, often relating to ‘anal stenosis’ and ‘rectal atresia’ excluded many potentially data-rich records.

## Conclusion

This review has demonstrated that more severe ARMs seem to be associated more frequently with a greater number of LSAs; this is the first study to systematically investigate this pattern of correlation. Considering the latest embryological evidence, our findings suggest that LSAs should be considered as a distinct set of defects to the genitourinary anomalies with which they are usually grouped. Most importantly, research into the long-term implications for patients with LSAs must be prioritised, in order to inform the multidisciplinary surgical management of ARM-LSA patients which considers the short-term control of faecal and urinary continence, as well as long-term sexual, gynaecological, cosmetic and psychosocial health.

## Supplementary Information

Below is the link to the electronic supplementary material.

Supplementary Material 1.

## Data Availability

No datasets were generated or analysed during the current study.

## Appendix: list of studies accepted for full text review

1. Abdel Aleem A, el Sheikh S, Mokhtar A, Ghafouri H, Saleem M (1985) The perineal groove and canal in males and females–a third look. Zeitschrift fur Kinderchirurgie : organ der Deutschen, der Schweizerischen und der Osterreichischen Gesellschaft fur Kinderchirurgie = Surgery in infancy and childhood 40:303–307.

2. AbouZeid AA, Mohammad SA, Sos MR, Guirguis NN, Mahmoud HA, El-Mahdy M (2022) Cloaca-Like Anomalies in the Male: A Report on Two Cases. European journal of pediatric surgery reports 10:e93–e97

3. Aihole JS (2022) A congenital long rectoscrotal fistula: A rare variant. Radiology Case Reports 17:3511–3514

4. Albers N, Ulrichs C, Glüer S, Hiort O, Sinnecker GH, Mildenberger H, Brodehl J (1997) Etiologic classification of severe hypospadias: implications for prognosis and management. The Journal of pediatrics 131:386–392

5. Aliefendioglu D, Bademci G, Keskil S, Somuncu S, Misirlioglu E, Cakmak AM (2007) VACTERL-H associated with central hypothyroidism: A case report. Genetic Counseling 18:331–335

6. Anagnostopoulos D, Valioulis I, Sfougaris D, Malliaropulos N, Foroglou P, Spyridakis G, Kallergis K (1993) Anatomic abnormalities of the genital tract in ano-rectal malformations. Bulletin de l’Association des anatomistes 77:5–7

7. Anonymous (2021) Corrigendum to: Persistence of mullerian duct structures in a genetic male with distal monosomy 10q (American Journal of Medical Genetics Part A, (2015), 167, 4, (791-796), 10.1002/ajmg.a.37014). American Journal of Medical Genetics, Part A 185:3176

8. Apold J, Dahl E, Aarskog D (1976) The Vater association: malformations of the male external genitalia. Acta paediatrica Scandinavica 65:150–152

9. Ari H, Sezer A, Ertas NBC, Donmez BO, Erdeve SS, Cetinkaya S (2022) A Case of Under virilized Male with 18q Partial Monosomy and 10p Duplication. Hormone Research in Paediatrics 95:565

10. Ayamba AM, Maalman RS-E, Donkor YO, Anyorigiya JN (2021) Complete Penoscrotal Transposition with Other Extragenital Anomalies in a Neonate Delivered at Term. Case reports in urology 2021:6676301–6676301

11. Banu T, Chowdhury TK, Hoque M, Rahman MA, Mushfiqur (2013) Cloacal malformation variants in male. Pediatric Surgery International 29:677

12. Banu T, Hoque M, Laila K, Ashraf Ul H, Hanif A (2009) Management of male H-type anorectal malformations. Pediatric surgery international 25:857–861

13. Bargaje A, Yerger JF, Khouzami A, Jones C (2008) Cloacal dysgenesis sequence. Annals of Diagnostic Pathology 12:62–66

14. Bawa M, Garge S, Sekhon V, Rao K (2015) Inguinal Ectopic Scrotum, Anorectal Malformation with Sacral Agenesis and Limb Defects: An Unusual Presentation. Journal of the Korean Association of Pediatric Surgeons 21:32–34

15. Baykara AS (2024) How to approach cases of anal atresia with rectoscrotal fistula: Low or intermediate? Turk J Pediatr Surg 38:027–030

16. Bendre P, Palse N, D’Souza F (2016) Cloaca with duplicated phallus - A case report. Journal of Pediatric Surgery Case Reports 10:32–34

17. Berry SA, Johnson DE, Thompson TR (1984) Agenesis of the penis, scrotal raphe, and anus in one of monoamniotic twins. Teratology 29:173–176

18. Boduroglu K, Alikasifoglu M, Tuncbilek E, Uludogan S (1998) Ring chromosome 13 in an infant with multiple congenital anomalies and penoscrotal transposition [1]. Clinical Dysmorphology 7:299–301

19. Browne D (1951) Some Congenital Deformities of the Rectum, Anus, Vagina and Urethra. Ann R Coll Surg Engl 8:173–192

20. Brunero M, Zilioli M, Falzoni PU, Sorrentino G, Balossini F, La Capria A (1996) [Recto-cutaneous penile fistula. A description of a clinical case and review of the literature]. Minerva pediatrica 48:393–396

21. Buyukkayhan D, Kurtoglu S, Koklu E, Hatipoglu N, Akcakus M (2007) Complete penoscrotal transposition associated with aortic stenosis in a newborn. Journal of pediatric endocrinology & metabolism : JPEM 20:881–881

22. Chadha R, Gupta S, Mahajan JK, Kothari SK (2001) Two cases of pseudoduplication of the external genitalia. Pediatric Surgery International 17:572–574

23. Chadha R, Kothari SK, Tanwar US, Gupta S (2001) Female pseudohermaphroditism associated with cloacal anomalies: faulty differentiation in the caudal developmental field. Journal of pediatric surgery 36:1

24. Chadha R, Sharma A, Bagga D, Mahajan JK (1998) Pseudoexstrophy associated with congenital pouch colon. Journal of Pediatric Surgery 33:1831–1833

25. Chatterjee SK, Basu AK, Chatterjee US (2005) Rectourinary perineal fistula - a unique anomaly. Journal of Pediatric Surgery 40:1658–1661

26. Chatterjee SK, Chatterjee US, Chatterjee U (2003) Perineal groove with penoscrotal hypospadias. Pediatric Surgery International 19:554–556

27. Chiaramonte RM, Rich MA, Brock WA (1992) Prolapsing congenital polyp of posterior urethra and urethral duplication associated with imperforate anus. Urology 40:522–524

28. Chu S-M, Ming Y-C, Chao H-C, Luo C-C (2009) An accessory labioscrotal fold associated with anorectal malformation in female neonates. Journal of pediatric surgery 44:E17–E19

29. Chung JL, Choi JR, Park MS, Choi SH (2001) A case of del(13)(q22) with multiple major congenital anomalies, imperforate anus and penoscrotal transposition. Yonsei Medical Journal 42:558–562

30. Currarino G (1986) Large prostatic utricles and related structures, urogenital sinus and other forms of urethrovaginal confluence. Journal of Urology 136:1270–1279

31. Currarino G (1994) Imperforate anus associated with a recto-bulbar-cutaneous fistula. Journal of Pediatric Surgery 29:102–105

32. De Volo CPB, Alfonsi M, Morizio E, Franchi PG, Stuppia L, Chiarelli F, Tumini S, Palka G, Calabrese G (2013) YQ microdeletion identified by array-CGH in a patient with vacterl association and peno-scrotal transposition. Chromosome Research 21:S119

33. Demehri FR, Lazow SP (2021) A novel anorectal malformation variant: Anocutaneous fistula presenting as median raphe abscesses. 57:718–720

34. Dewhurst CJ (1963) Gynaecological Disorders of Infants and Children, 1st ed. Casell & Company Ltd., London

35. Dewhurst CJ (1968) Congenital Malformations of the Genital Tract in Childhood. The Journal of Obstetrics and Gynaecology of the British Commonwealth 75:377–391

36. Dewhurst J (1978) Congenital malformations of the lower urinary tract. Clinics in Obstetrics and Gynaecology 5:51–65

37. Digray NC, Mengi Y, Goswamy HL, Thappa DR (1999) Congenital urethral anomalies in ano-rectal malformations. JK Practitioner 6:116–117

38. Donaldson GL, Bury RG (1982) Multiple congenital abnormalities in a newborn boy associated with maternal use of fluphenazine enanthate and other drugs during pregnancy. Acta Paediatrica Scandinavica 71:335–338

39. Duarte ML, Dos Santos LR, Da Silva ADQP, Da Silva MDQP (2021) Diphallia: a case report of a rare anomaly evaluated by magnetic resonance imaging. The Turkish Journal of Pediatrics 63:917–921

40. Dubowitz V (1962) Virilisation and Malformation of a Female Infant. The Lancet 280:405–406

41. Endo M, Hayashi A, Ishihara M, Maie M, Nagasaki A, Nishi T, Saeki M (1999) Analysis of 1,992 Patients With Anorectal Malformations Over the Past Two Decades in Japan. Journal of Pediatric Surgery 34:435–441

42. Escobar LF, Weaver DD, Bixler D, Hodes ME, Mitchell M (1987) Urorectal Septum Malformation Sequence. American Journal of Diseases of Children 141:1021–1024

43. Faria JAD, Loch Batista R, Gomes NLRA, Moraes DR, Nishi MY, Zanardo E, Kulikowski L, Costa EMF, Mendonca BB, Domenice S (2018) Copy number variants in syndromic patients with disorders of sex development. Endocrine Reviews 39:

44. Fernandez JAF, Hueck LP, Fermin JC (2014) Exstrophy of rectal duplication associated with anorectal malformation and penoscrotal transposition with perineal hypospadias. A case report. Investigacion Clinica (Venezuela) 55:168–172

45. Ferro F, Lais A, Korman R, Caterino S (1991) Accessory perineal scrotum associated with anorectal malformation. A report of 2 cases. South African journal of surgery Suid-Afrikaanse tydskrif vir chirurgie 29:32–34

46. Fitzgerald RJ, Watters K, Bissett WH, Bjordal R, Monclair T (2002) Translevator anal anomalies with cutaneous fistulae passing deep to the scrotum. Journal of Pediatric Surgery 37:1326–1329

47. Fryns JP, Verresen H, Van den Berghe H (1997) Prenatal growth retardation, microphthalmos/iris coloboma, cloudy cornea, urogenital anomalies and microcephaly. A possible new sublethal syndrome. Clinical Genetics 51:164–166

48. Gao Y (2020) Fetal hypospadias with imperforate anus: Case report. Chinese Journal of Medical Imaging Technology 36:1920

49. Ghritlaharey RK, Malik R (2009) Split notochord syndrome with dorsal enteric fistula in a male neonate: A case report. Journal of Clinical and Diagnostic Research 3:1797–1800. https://doi.org/10.1002/(SICI)1096-9926(199802)57:270::AID-TERA53.0.CO;2-A

50. Günaydin M, Aritürk E, Rizalar R, Görk S, Bernay F, Gürses N (1997) Unilateral inguinal ectopic scrotum and imperforate anus: a case report. Journal of pediatric surgery 32:1360–1361

51. Gupta DK, Charles AR, Srinivas M (2003) Exstrophy Variants: Should they be Considered Malformation Complexes Separate from Classic Exstrophy? European Journal of Pediatric Surgery 13:377–382

52. Gupta RK, Tiwari P, Parelkar SV, Sanghvi BV, Mudkhedkar KP, Mhaskar SS, Shah RS (2022) An Unusual Case of Type A Posterior Cloaca Associated with 46XX Disorder of Sexual Differentiation with Y Duplication of Urethra. Journal of Indian Association of Pediatric Surgeons 27:251–254

53. Harris KT, Pique DG, Wehrli LA, Trecartin A, Roach J, Meeks NJ, Nokoff NJ, Wilcox DT, Bischoff A (2023) Anorectal malformation in a 46,XY patient with a de novo stop-loss variant in PPP1R12A and associated difference in sexual development: A case report. Journal of Pediatric Surgery Case Reports 95:102679

54. Hashizume N, Asagiri K, Fukahori S, Komatsuzaki N, Yagi M (2018) Perineal lipoma with anorectal malformation: Report of two cases and review of the literature. Pediatrics International 60:83–85

55. Hendren WH (1992) Cloacal malformations: Experience with 105 cases. Journal of Pediatric Surgery 27:890–901

56. Hersh JH, Jaworski M, Solinger RE, Weisskopf B, Donat J (1986) Townes syndrome. A distinct multiple malformation syndrome resembling VACTERL association. Clinical pediatrics 25:100–102

57. Hicks CM, Skoog SJ, Done S (1989) Ectopic vas deferens, imperforate anus and hypospadias: A new triad. Journal of Urology 141:586–588

58. Hollowell JG, Witherington R, Ballagas AJ, Burt JN (1977) Embryologic considerations of diphallus and associated anomalies. The Journal of urology 117:728–732

59. Hongying W, Chao H, Li L, Yinghua L, Ting W, Yuxin Z, Haibo L (2019) Clinical and genetic analysis of a child with chromosomal 13q32. l-q33. 3 deletion. Chinese Journal of Medical Genetics 36:1213–1218

60. Huang H, Liu X, Li Z, Lin J, Yang H, Xu Z (2023) Ectopic scrotum and penoscrotal transposition: Case report and literature review. Frontiers in Pediatrics 11:1015384

61. Inde Y, Terada Y, Ikegami E, Sekiguchi A, Nakai A, Takeshita T (2014) Bifid scrotum and anocutaneous fistula associated with a perineal lipomatous tumor complicated by temporary bilateral cryptorchidism in utero mimicking ambiguous genitalia: 2-D/3-D fetal ultrasonography. The journal of obstetrics and gynaecology research 40:843–848

62. Jadhav B, Rijhwani A (2018) Anorectal Atresia with Rectoscrotal Fistula: A Rare Anorectal Malformation. Journal of Neonatal Surgery 7:13

63. Jaiman S (2020) External ear malformations and their association with multiple congenital anomalies on perinatal autopsy. Pediatric and Developmental Pathology 23:492–493

64. Jain A, Lone Y (2023) Urethral duplication associated with ectopic scrotum: A case study. Journal of Indian Association of Pediatric Surgeons 28:348–351

65. Jam M, Shahzad ZA, Mehmood T, et al (2013) Unusual Sacrococcygeal Teratoma (SCT) with accessory penis, anorectal malformation, bifid scrotum, bilateral inguinal hernias and hypospadias. Pakistan Journal of Medical and Health Sciences 7:1224–1226

66. Jun LH, Jacobsen A, Rai R (2021) Case Report: A Case Series of Rare High-Type Anorectal Malformations With Perineal Fistula: Beware of Urethral Involvement. Frontiers in Surgery 8:1–6

67. Kaneko Y, Ikeuchi T, Sasaki M, Stakae Y, Kuwajima S (1975) A male infant with monosomy 21. HUMANGENETIK 29:1–7

68. Kang HS, Yang A, Haynes J, Lange P, Oiticica C (2023) Anorectal malformation with rectal-urethral-scrotal fistula: A case series. Journal of Pediatric Surgery Case Reports 99:102731

69. Karaca I, Turk E, Ucan AB, Yayla D, Itirli G, Ercal D (2014) Surgical management of complete penile duplication accompanied by multiple anomalies. Canadian Urological Association journal = Journal de l’Association des urologues du Canada 8:E741–E743

70. Kayima P, Kitya D, Punchak M, Anderson GA, Situma M (2019) Patterns and treatment outcomes of anorectal malformations in Mbarara Regional Referral Hospital, Uganda. Journal of Pediatric Surgery 54:838–844

71. Kuhnle U, Bartsch O, Werner W, Schuster T (2000) Penoscrotal inversion, hypospadias, imperforate anus, facial anomalies, and developmental delay: definition of a new clinical syndrome. Pediatric Surgery International 16:396

72. Kumar V, Apte AV, Gangopadhyay AN, Singh S (2004) Tracheoesophageal fistula and amastia with other anomalies: an unusual association. Pediatric Surgery International 20:378

73. Kyrklund K, Taskinen S, Rintala RJ, Pakarinen MP (2016) Sexual Function, Fertility and Quality of Life after Modern Treatment of Anorectal Malformations. The Journal of urology 196:1741–1746

74. Lacassie Y, McKusick VA (1975) The G syndrome: analysis of 2 new cases, New Chromosomal and Malformation Syndromes. Birth Defects, Origartser 11:334

75. Lena F, Pellegrino C, Zaccara AM, et al (2022) Anorectal malformation, urethral duplication, occult spinal dysraphism (ARM-UD-OSD): a challenging uncommon association. Pediatric Surgery International 38:1487–1494

76. Lowsley OS (1948) Persistent Cloaca in the Female: Report of Two Cases Corrected by Operation. The Journal of Urology 59:692–707

77. Lutavi JB, Mahugi MR, Daudi K, Nicodem K, Robert M (2022) Complete penoscrotal transposition with multiple congenital malformations. Journal of Clinical Case Reports, Medical Images and Health Sciences 1:

78. Mandell J, Bromley B, Peters CA, Benacerraf BR (1995) Prenatal sonographic detection of genital malformations. Journal of Urology 153:1994–1996

79. Mansouri MR, Carlsson B, Davey E, Nordenskjöld A, Wester T, Annerén G, Läckgren G, Dahl N (2006) Molecular genetic analysis of a de novo balanced translocation t(6;17)(p21.31;q11.2) associated with hypospadias and anorectal malformation. Human genetics 119:162–168

80. Mazoochi M, Faraji-Dana H, Namazi H, Mohammadian Khonsari N, Jamshidi F (2022) A case report of complete penoscrotal transposition with hypospadias and imperforates anus. An ethical dilemma. Journal of Neonatal Nursing 28:134–136

81. Mendoza-Londono R, Lammer E, Watson R, et al (2005) Characterization of a new syndrome that associates craniosynostosis, delayed fontanel closure, parietal foramina, imperforate anus, and skin eruption: CDAGS. American Journal of Human Genetics 77:161–168

82. Mirshemirani A, Roshanzamir F, Shayeghi S, Mohajerzadeh L, Hasas-Yeganeh S (2010) Diphallus with imperforate anus and complete duplication of recto-sigmoid colon and lower urinary tract. Iranian journal of pediatrics 20:229–232

83. Mirshemirani A-R, Sadeghyian N, Mohajerzadeh L, Molayee H, Ghaffari P (2010) Diphallus: Report on Six Cases and Review of the Literature. Iranian Journal of Pediatrics 20:353–357

84. Misra D, Mushtaq I, Drake DP, Kiely EM, Spitz L (1996) Associated urologic anomalies in low imperforate anus are capable of causing significant morbidity: A 15-year experience. Urology 48:281–283

85. Mittal A, Airon RK, Magu S, Rattan KN, Ratan SK (2004) Associated Anomalies with Anorectal Malformation. Indian Journal of Pediatrics 71:509–514

86. Mortazavi F, Aslanabadi S, Mahnama ST (2007) Urogenital anomalies associated with anorectal malformations. Pakistan Journal of Medical Sciences 23:172–175

87. Mukhtar RA, Baskin LS, Stock PG, Lee H (2009) Long-term survival and renal transplantation in a monozygotic twin with cloacal dysgenesis sequence. Journal of pediatric surgery 44:e31–e33

88. Mukunda R, Bendre PS, Redkar RG, Hambarde S (2010) Diphallus with anorectal malformation-case report. Journal of pediatric surgery 45:632–634

89. Munyali A, Buhendwa C, Ganywamulume B, Gloire B, Ndanda L, Longombe AO (2025) Adolescent anorectal malformations: Case series about 3 cases. International Journal of Surgery Case Reports 127:110913 https://doi.org/10.1016/j.ijscr.2025.110913

90. Nakano Y, Aizawa M, Honma S, Osa Y (2009) Completely separated scrotum and vesicointestinal fistula without exstrophy as a novel manifestation of aphallia: a case report. Urology 74:1303–1305

91. Neri G, Blumberg B, Miles PV, Opitz JM (1984) Sensorineural deafness in the FG syndrome: Report on four new cases. American Journal of Medical Genetics 19:369–377

92. Omil-Lima D, Gupta K, Prunty M, Miyasaka EA, Joyce EL, Nguyen C, Hannick JH (2021) Bladder Agenesis and Bilateral Ectopic Ureters in an Infant Male With Cystic Renal Dysplasia, Imperforate Anus, and Penoscrotal Transposition. Urology 156:256–259

93. Ozcan C, Celik A, Erdener A (1999) Accessory perineal scrotum associated with anorectal malformation. BJU international 83:729–730

94. Pandit SK, Solanki RS, Budhiraja S, Rattan KN (1999) Prepenile scrotum with high anorectal malformation. Indian pediatrics 36:938–939

95. Paria P (2015) Complete penoscrotal transposition. Journal of Pediatric Sciences 7:e247 https://doi.org/10.17334/jps.54342

96. Parida SK, Hall BD, Barton L, Fujimoto A (1995) Penoscrotal transposition and associated anomalies: Report of five new cases and review of the literature. American Journal of Medical Genetics 59:68–75

97. Puri A, Choudhury S, Yadav P, Grover J, Pant N, Chadha R (2011) Congenital pouch colon and segmental dilatation of the colon: A report of two unusual cases. Journal of Indian Association of Pediatric Surgeons 16:61

98. Ramos FJ, McDonald-McGinn DM, Emanuel BS, Zackai EH (1992) Tricho-Rhino-Phalangeal syndrome type II (Langer-Giedion) with persistent cloaca and prune belly sequence in a girl with 8q interstitial deletion. American Journal of Medical Genetics 44:790–794

99. Ramzisham ARM, Thambidorai CR (2005) Perineal hamartoma with accessory scrotum, anorectal anomaly, and hypospadias - A rare association with embryological significance: Case report. Pediatric Surgery International 21:478–481

100. Rattan KN, Pandit SK, Budhiraja S (1997) Accessory scrotum with imperforate anus. Pediatric Surgery International 12:217

101. Redman JF, Moore JM (1979) Congenital rectourethral fistula with hypospadias and five ureters. Urology 13:186–187

102. Reilly W, Hinman F, Pickering D, Crane J (1958) Phallic urethra in Female Pseudohermaphroditism. AMA Am J Dis Child 95:9–17

103. Reppucci ML, Alaniz VI, Wehrli LA, de La Torre L, Wood D, Wilcox DT, Appiah LC, Pena A, Bischoff A (2023) Reproductive and Family Building Considerations for Female Patients with Anorectal And Urogenital Malformations. Journal of Pediatric Surgery 58:1450–1457

104. Rich MA, Brock WA, Peña A (1988) Spectrum of genitourinary malformations in patients with imperforate anus. Pediatric surgery international 3:110–113

105. Rodriguez JI, Palacios J, Omenaca F, Lorente M (1991) Polyasplenia, caudal deficiency, and agenesis of the corpus callosum. American Journal of Medical Genetics 38:99–102

106. Rohrer L, Vial Y, Gengler C, Tenisch E, Alamo L (2020) Prenatal imaging of anorectal malformations - 10-year experience at a tertiary center in Switzerland. Pediatric Radiology 50:57–67

107. Rump P, Hordijk R, De Raad M, Niessen RC (2010) Novel MED12 mutation in Opitz-Kaveggia syndrome. Genetic Counseling 21:121–122

108. Samuk I, Bischoff A, Hall J, Levitt M, Pena A (2016) Anorectal malformation with rectobladder neck fistula: A distinct and challenging malformation. Journal of Pediatric Surgery 51:1592–1596

109. Sankar VH, Phadke SR (2006) Ring Chromosome 13 in an Infant with Ambiguous Genitalia. Indian Pediatrics 43:258–260

110. Schrander J, Schrander-Stumpel C, Berg J, Frias JL (1995) Opitz BBBG syndrome: New family with late-onset, serious complication. Clinical Genetics 48:76–79

111. Seller MJ (1990) Twins Discordant for Cleft Lip and Palate. American Journal of Medical Genetics 37:530–531

112. Sesenna R (1959) [On a complex urogenital malformation (persistent cloaca, hypospadias, renal aplasia)]. Urologia internationalis 8:317–335

113. Sfoungaris D, Mouravas V, Lambropoulos V, Kepertis C, Spyridakis I (2016) Imperforate Anus with Fistula Exiting at the Penile Skin. Journal of clinical and diagnostic research : JCDR 10:PD01–PD02

114. Shandilya G, Pandey A, Pant N, Singh G, Kumar A, Rawat J (2022) Evaluation and management of “low” anorectal malformation in male children: an observational study. Pediatric Surgery International 38:337–343

115. Sharma AK, Goel D, Kothari SK (1999) Perineal-mound defects. Pediatric Surgery International 15:227–229

116. Sharma SP, Upadhyaya VD, Pandey A, Gangopadhyay AN (2008) Rectal atresia with rectolabial fistula. Journal of Indian Association of Pediatric Surgeons 13:75–76

117. Shono T, Suita S, Kubota M, Ariga H, Taguchi T, Satoh S, Nakano H, Watanabe T (1996) Prenatal ultrasonograms showing a reduced amniotic cavity in a patient with multiple genitourinary, anorectal, and limb anomalies. Pediatric Surgery International 11:406–407

118. Shoshany G, Gottfied E, Bar-Maor JA (1996) Accessory scrotum and anorectal malformation associated with “pseudo” prune belly in a neonate. Journal of perinatology : official journal of the California Perinatal Association 16:224–226

119. Singh RR, Seager RL, Shibu M, Misra D, Joshi A (2020) Ectopic scrotum: Single stage rotational flap reconstruction with orchidopexy. Journal of Pediatric Surgery Case Reports 58:101469

120. Singh S, Ahmed I, Rawat J, Panday A (2011) Association of anorectal malformation with duplicated colon, sacral meningomyelocele and scrotal anomalies. BMJ Case Reports 2011:n/a

121. Smyth A, Nowik C, Pugash D, Rosenbaum D (2019) Peeing double-A case report of caudal duplication. Pediatric Radiology 49:S149

122. Snyder WH (1966) Some unusual forms of imperforate anus in female infants. The American Journal of Surgery 111:319–325

123. So EP, Brock W, Kaplan GW (1980) Ectopic labium and VATER association in a newborn. The Journal of urology 124:156–157

124. Somuncu S, Ariturk E, Gunaydin M, Rizalar R, Bernay F, Gurses N (1996) Rare types of anorectal malformation: Report of three cases. Pediatrik Cerrahi Dergisi 10:36–39

125. Stephens FD (1963) Congenital Malformations of the Rectum, Anus and Genito-Urinary Tracts, 1st ed. E & S Livingstone Ltd, Edinburgh

126. Subbarao P, Bhatnagar V, Mitra DK (1994) The association of sacrococcygeal teratoma with high anorectal and genital malformations. Australian and New Zealand Journal of Surgery 64:214–215

127. Tabari AK, Mirshemirani A, Tabari NK (2012) Complete colonic duplication in children. Caspian Journal of Internal Medicine 3:436–439

128. Taghavi K, Trachta J, Mushtaq I (2019) Urethral duplication: A case for careful examination. Archives of Disease in Childhood 104:685

129. Takahashi M, Kanamori Y, Tanaka H, Watanabe T, Sato K, Ohno M, Yamada W, Yamada K, Takezoe T, Fuchimoto Y (2014) Accessory scrotum complicated with perineal lipoblastoma and anorectal malformation - An extremely rare phenotype of the anomalies. Journal of Pediatric Surgery Case Reports 2:350–352

130. Townes PL, Dehart GK, Ziegler NA (1964) Trisomy 17-18—An Evaluation of Preconceptional Parental Irradiation as a Possible Etiologic Factor. The Journal of pediatrics 65:870–879

131. Tsai FJ, Lin JS, Tsai CH (1992) G syndrome: report of two cases. Zhonghua Minguo xiao er ke yi xue hui za zhi [Journal] Zhonghua Minguo xiao er ke yi xue hui 33:218–225

132. Tumini S, Alfonsi M, Carinci S, et al (2019) Yq Microdeletion in a Patient with VACTERL Association and Shawl Scrotum with Bifid Scrotum: A Real Pathogenetic Association or a Coincidence? Cytogenetic and Genome Research 158:121–125

133. van Rooij IALM, van der Zanden LFM, Bongers EMHF, et al (2016) AGORA, a data‐ and biobank for birth defects and childhood cancer. Birth Defects Research Part A: Clinical and Molecular Teratology 106:675–684

134. Vela Sampedro I, Palacios Marques AM, Blasco Medina S, Quijada Cazorla MA, Martinez Escoriza JC (2019) Congenital perineal hamartoma with genital and anorectal malformation associated with genetic alteraction: Duplicity of the TGIF2LX gene. Progresos de Obstetricia y Ginecologia 62:156–158

135. Verloes A, Gillerot Y, Delfortrie J, Zeevaert-Arnold MT, Collard R, Koulischer L, Fryns JP (1990) Male pseudohermaphroditism with persistent müllerian structures, mental retardation and Borjeson-Forssman-Lehmann-like features: a new syndrome? Genetic counseling (Geneva, Switzerland) 1:219–225

136. Villarreal DH, Ortiz VN, Iturregui JR, Suarez G, Duran N (1998) Bifid scrotum, perineal hamartoma and high imperforate anus: a case report. Boletin de la Asociacion Medica de Puerto Rico 90:93–94

137. Voigt HR, Wentzel SW (2005) Complete duplication of the bladder, urethra and external genitalia in a male neonate with an imperforate anus. International journal of urology : official journal of the Japanese Urological Association 12:702–704

138. Wahyudi I, Deswanto IA, Situmorang GR, Rodjani A (2019) One stage rotation flap scrotoplasty and orchidopexy for the correction of ectopic scrotum: A case report. Urology case reports 25:100886–100886

139. Walsh LE, Vance GH, Weaver DD (2001) Distal 13q deletion syndrome and the VACTERL association: Case report, literature review, and possible implications. American Journal of Medical Genetics 98:137–144

140. Wang K, Peng C, Pang W, Wang Z, Wu D, Zhang D, Siyin ST, Chen Y (2021) Anorectal Malformations Associated With Labioscrotal Fold Malformation and Perineal Mass in Pediatric Patients: Over a Decade of Experience. Frontiers in pediatrics 9:627188–627188

141. Wang YF, Chou HC, Chen CY, Tsao PN (2020) A neonate with imperforate anus and accessory scrotum with scrotal bifida. Journal of the Formosan Medical Association 119:1331–1332

142. Wenstrup RJ, Pagon RA (1985) Female pseudohermaphroditism with anorectal, mullerian duct, and urinary tract malformations: Report of four cases. The Journal of Pediatrics 107:751–754

143. Wese FX, Claus D, Hennebert P, Kayumbi J, Otte JB (1983) [Anomalies of the urogenital system associated with anorectal malformations]. Acta chirurgica Belgica 82:178–182

144. Wu W-H, Chuang J-H, Ting Y-C, Lee S-Y, Hsieh C-S (2002) Developmental anomalies and disabilities associated with hypospadias. The Journal of urology 168:229–232
