# Supplementary material for: ARM in ARM? Investigating the co-occurrence of anorectal malformations and labioscrotal anomalies
Source: Pediatr Surg Int. 2026 Jul 17;42(1):308. doi: 10.1007/s00383-026-06475-7 (PMC13379451; doi:10.1007/s00383-026-06475-7)
Supplement: Supplementary file 1 — Supplementary Material 1. [file 383_2026_6475_MOESM1_ESM.docx]

**Supplementary Materials for:**

ARM in ARM? Investigating the co-occurrence of anorectal malformations and labioscrotal anomalies

Patrick G G Sharman^1^*, Maria Randazzo, Ian Jones, Ingo Jester

**For submission to *Pediatric Surgery International***

^1^College of Medicine and Health, University of Birmingham, Edgbaston, B15 2TT

*Corresponding author. Email: pgs000@student.bham.ac.uk

**Supplementary Table 1** Anatomy and embryology of labioscrotal anomalies

| Anomaly | Morphological characteristics | Embryology of defect |
| --- | --- | --- |
| Scrotal anomalies | | |
| Bifid scrotum | Two halves of scrotum partially separated  Persistence of midline scrotal raphe separates two halves  Often associated with penoscrotal transposition and proximal hypospadias | Correlation between bifid scrotum and androgen insensitivity suggests defective androgen receptor gene (*AR Xq11-12*) may be responsible [1] |
| Ectopic scrotum | Additional hemiscrotum located along pathway of normal testicular descent [2]  Only occurs unilaterally  Contains testis | May be due to disruption of gubernaculum development  Alternative mechanical aetiology proposed by Singh *et al.*: limb buds physically keep labioscrotal folds medial, but absence of these permits lateral deviation, into ectopic position [3] |
| Accessory scrotum | Appears as additional epithelialised, rugated mass, usually on perineum [4]  Distinguished from ectopic scrotum by lack of testicular tissue  Diagnosis can only be confirmed with histological testing | Uncertain pathogenesis, possibly due to mesenchyme interrupting one labioscrotal swelling, dividing it into two, part of which develops as accessory scrotum [5] |
| Hemiscrotum | In normal anatomy, hemiscrotum refers to two bilateral halves of scrotum, separated by midline raphe  In context of LSAs, refers to complete separation of two hemiscrotum halves, with midline scrotal raphe absent | Severe form of bifid scrotum, where early arrest in fusion of labioscrotal swellings prevents formation of single scrotum [6] |
| Penoscrotal fusion | Poorly documented, sometimes referred to as ‘webbed penis’  Skin fold extends superiorly, tethers ventral aspect of penis to scrotum | Uncertain embryogenesis [7] |
| Penoscrotal transposition | Cephalo-caudal transposition of penis and scrotum, so penis lies between two hemiscrota (also causing bifid scrotum), or is completely inferior to scrotum [8] | Early fusion of labioscrotal swellings before completion of caudal migration may be responsible  Abnormal positioning of genital tubercle (precursor to penis) may be aetiological factor |
| Scrotal raphe anomaly | Spectrum of defects associated with abnormal medial fusion of labioscrotal swellings  May present as:   - *Prominent median raphe* – ridge-like appearance - *Deviated raphe* – lateral deviation - *Absent median raphe* – associated with pathological hemiscrotum - *Split median raphe* – bilateral, paramedian ridges | Various aberrations of median mesodermal and overlying ectodermal components [6] |
| Scrotoschisis | Defect in wall of scrotum permits herniation of testis  Otherwise scrotum appears normal | Uncertain embryogenesis |
| (Hemi)scrotal agenesis/hypoplasia | Hypoplasia of scrotum may be unilateral (hemiscrotal hypoplasia) or bilateral (scrotal hypoplasia)  Scrotum without testis is usually hypoplastic  Characterised by smooth, non-rugated skin below penis  Midline raphe usually still present  Single hemiscrotum present on contralateral side in unilateral scrotal agenesis | Both hypoplasia and agenesis may be due to deficiency or absence of 5-α reductase, or partial/complete androgen insensitivity [9] |
| Proximal hypospadias | Severe form of ectopic urethral meatus  Can be classified into penoscrotal (least severe), scrotal and perineal (most severe) [10] | Endodermal urethral folds fuse in caudal-cephalic direction; early interruption of this results in proximal hypospadias  Underlying mechanism uncertain; known to have multifactorial causes [11] |
| Labial anomalies | | |
| Labial hypoplasia | May be unilateral or bilateral  May appear as partial hypoplasia, where anterior two-thirds are normal, but posterior third is hypoplastic or absent | Uncertain embryogenesis |
| Labial hypertrophy | May be unilateral or bilateral  Appears as abnormally prominent swellings | Associated with congenital adrenal hyperplasia, when excessive 5-α reductase stimulates increased production of dihydrotestosterone, causing hypertrophic growth of labia majora [12] |
| Labial fusion | Fusion of labia minora, which arise from urogenital folds  Appears as adherence of labia minora, sealing off vaginal orifice  Urethral meatus may also be covered [13] | Associated with congenital adrenal hyperplasia, in which urogenital folds fuse |
| Accessory labial fold | Additional fold of skin associated with labia majora | Proposed embryogenesis similar to accessory scrotum: interrupting mesenchymal tissue splits one labioscrotal fold as it migrates, resulting in additional labium [9, 14] |

**Supplementary Table 2** Terms used for search strategy [6, 9, 15, 16]

| ARM-related search terms | LSA-related search terms |
| --- | --- |
| Ano-rectal malformation | Labioscrotal |
| Anorectal fistula | Labio-scrotal |
| Ano-rectal fistula | Bifid scrotum |
| Rectoperineal fistula | Accessory scrotum |
| Recto-perineal fistula | Ectopic scrotum |
| Rectourethral fistula | Hemiscrotum |
| Recto-urethral fistula | Penoscrotal fusion |
| Rectovestibular fistula | Scrotal transposition |
| Recto-vestibular fistula | Scrotal raphe anomaly |
| Rectobulbar fistula | Scrotoschisis |
| Recto-bulbar fistula | Scrotal agenesis |
| Rectoprostatic fistula | Scrotal hypoplasia |
| Recto-prostatic fistula | Hemiscrotal agenesis |
| Rectovaginal fistula | Hemiscrotal hypoplasia |
| Recto-vaginal fistula | Hypospadias |
| Rectobladder fistula | Labial hypoplasia |
| Recto-bladder fistula | Labial hypertrophy |
| Rectovesical fistula | Labial fusion |
| Recto-vesical fistula | Accessory labial fold |
| Imperforate anus | Penoscrotal |
| Cloaca |  |
| Cloacal exstrophy |  |
| Anal stenosis |  |
| Rectal stenosis |  |
| Rectal atresia |  |
| Pouch colon |  |
| H fistula |  |
| Cutaneous fistula |  |
| ARM |  |

**Supplementary Fig. 1** Difference between proportions of ARM cases with a single vs multiple LSAs

This chart further exemplifies the trend shown by Figure 2 in the main text. This chart shows the respective differences between the proportions of patients with a single LSA associated vs those with multiple associated, for each of the three groupings of ARMs (Table 1 in main text). As the severity of ARM increases, the difference between the proportion of cases associated with a single vs multiple LSAs decreases. This indicates that as ARM type becomes more severe, the likelihood that only a single LSA will be associated decreases - and therefore the proportion associated with multiple LSAs increases.

## References

1. Swartz JM, Ciarlo R, Denhoff E, et al (2017) Variation in the clinical and genetic evaluation of undervirilized boys with bifid scrotum and hypospadias. J Pediatr Urol 13:293.e1-293.e6. https://doi.org/10.1016/j.jpurol.2017.01.004

2. Bawa M, Garge S, Sekhon V, Rao K (2015) Inguinal Ectopic Scrotum, Anorectal Malformation with Sacral Agenesis and Limb Defects: An Unusual Presentation. J Korean Assoc Pediatr Surg 21:32–34. https://doi.org/10.13029/jkaps.2015.21.2.32

3. Singh RR, Seager RL, Shibu M, et al (2020) Ectopic scrotum: Single stage rotational flap reconstruction with orchidopexy. J Pediatr Surg Case Rep 58:101469. https://doi.org/10.1016/j.epsc.2020.101469

4. Zhong L, Zou X, Sun J (2020) Two Cases of Accessory Scrotum with Soft Fibroma in Neonates. Indian J Surg 82:1271–1272. https://doi.org/10.1007/s12262-020-02241-8

5. Sule JD, Skoog SJ, Tank ES (1994) Perineal lipoma and the accessory labioscrotal fold: an etiological relationship. J Urol 151:475–477. https://doi.org/10.1016/s0022-5347(17)34996-0

6. Fahmy MAB (2022) Congenital Scrotal Anomalies. In: Normal and Abnormal Scrotum. Springer International Publishing, Cham, pp 117–119

7. Bonitz RP, Hanna MK (2016) Correction of congenital penoscrotal webbing in children: A retrospective review of three surgical techniques. J Pediatr Urol 12:161.e1-161.e5. https://doi.org/10.1016/j.jpurol.2016.02.003

8. Fahmy MAB, El Shennawy AAA, Edress AM (2014) Spectrum of penoscrotal positional anomalies in children. Int J Surg 12:983–988. https://doi.org/10.1016/j.ijsu.2014.08.001

9. Fahmy MAB (2015) Rare Congenital Genitourinary Anomalies, 1st ed. Springer Berlin Heidelberg, Berlin, Heidelberg

10. Baskin LS, Ebbers MB (2006) Hypospadias: anatomy, etiology, and technique. J Pediatr Surg 41:463–472. https://doi.org/10.1016/j.jpedsurg.2005.11.059

11. Halaseh SA, Halaseh S, Ashour M (2022) Hypospadias: A Comprehensive Review Including Its Embryology, Etiology and Surgical Techniques. Cureus. https://doi.org/10.7759/cureus.27544

12. Siddiqui SA, Soomro N, Ganatra A (2012) Classic congenital adrenal hyperplasia: A delayed presentation. Pak J Med Sci 29:. https://doi.org/10.12669/pjms.291.2830

13. Srivastava S, Pandey A, Kumar P, et al (2021) Management of labial adhesion in a developing country—an observational study. Egypt Pediatr Assoc Gaz 69:41. https://doi.org/10.1186/s43054-021-00089-5

14. Chu S-M, Ming Y-C, Chao H-C, Luo C-C (2009) An accessory labioscrotal fold associated with anorectal malformation in female neonates. J Pediatr Surg 44:E17–E19. https://doi.org/10.1016/j.jpedsurg.2008.12.028

15. Docimo SG, Canning D, Khoury A, Salle JLP (2018) The Kelalis--King--Belman Textbook of Clinical Pediatric Urology, 6th ed. CRC Press

16. Radmayr C, Bogaert G, ’t Hoen LA, et al (2025) EAU Pediatric Urology Guidelines. European Association of Urology, Arnhem, The Netherlands
